# Cervical Sensorimotor Function Tests Using a VR Headset—An Evaluation of Concurrent Validity

**DOI:** 10.3390/s24175811

**Published:** 2024-09-07

**Authors:** Karin Forsberg, Johan Jirlén, Inger Jacobson, Ulrik Röijezon

**Affiliations:** Department of Health, Education, and Technology, Luleå University of Technology, 97187 Luleå, Sweden; johan.jirlen@ltu.se (J.J.); inger.jacobson@ltu.se (I.J.); ulrik.roijezon@ltu.se (U.R.)

**Keywords:** agreement, correlation, neck pain, joint position sense, reaction time, sensorimotor, velocity, virtual reality, VR

## Abstract

Sensorimotor disturbances such as disturbed cervical joint position sense (JPS) and reduced reaction time and velocity in fast cervical movements have been demonstrated in people with neck pain. While these sensorimotor functions have been assessed mainly in movement science laboratories, new sensor technology enables objective assessments in the clinic. The aim was to investigate concurrent validity of a VR-based JPS test and a new cervical reaction acuity (CRA) test. Twenty participants, thirteen asymptomatic and seven with neck pain, participated in this cross-sectional study. The JPS test, including outcome measures of absolute error (AE), constant error (CE), and variable error (VE), and the CRA test, including outcome measures of reaction time and maximum velocity, were performed using a VR headset and compared to a gold standard optical motion capture system. The mean bias (assessed with the Bland–Altman method) between VR and the gold standard system ranged from 0.0° to 2.4° for the JPS test variables. For the CRA test, reaction times demonstrated a mean bias of −19.9 milliseconds (ms), and maximum velocity a mean bias of −6.5 degrees per seconds (°/s). The intraclass correlation coefficients (ICCs) between VR and gold standard were good to excellent (ICC 0.835–0.998) for the JPS test, and excellent (ICC 0.931–0.954) for reaction time and maximum velocity for the CRA test. The results show acceptable concurrent validity for the VR technology for assessment of JPS and CRA. A slightly larger bias was observed in JPS left rotation which should be considered in future research.

## 1. Introduction

Neck pain is a common musculoskeletal condition [1,2,3] causing extensive burden for individuals [4,5] and societies [3,6,7]. Neck pain can occur due to physical trauma to the head or neck, often referred to as whiplash trauma or whiplash-associated disorder (WAD) [8,9], or in more insidious ways, e.g., due to repetitive or prolonged unhealthy postures or movements [10,11,12].

Many people recover from neck pain disorder, but for a considerably large group of people the problem becomes recurrent or persistent over time [8,13,14]. The mechanisms behind recurrent or persistent neck pain disorders are not fully understood, but research has reported disturbances in sensorimotor control functions as a possible factor [15,16,17,18,19,20,21,22,23,24].

Sensorimotor control involves the sensory systems, including proprioception, processes within the central nervous system, and motor commands. This system is crucial for humans’ ability to maintain posture, stability, and control of movements [25]. Proprioception can be defined as the conscious and unconscious awareness of the joints and body parts’ position, their movements, and forces acting on the body [19]. In the cervical region, proprioception plays a crucial part for sensorimotor control due to the large number of proprioceptors in the cervical spine and their unique links to the vestibular and visual system [19,26].

Several sensorimotor function disturbances have been reported in people with neck pain such as reduced cervical joint position sense [27,28,29,30,31,32], movement sense [22,33,34,35], force sense [36,37], and a reduced ability to perform fast and precise cervical movements [15,35,38,39,40,41].

Assessment of sensorimotor functions, including proprioception, is essential to identifying disturbances, to guiding tailored precise interventions, and for evaluation of treatment effects [32,35,42]. Objective assessment of sensorimotor functions requires some kind of measurement system, most often found in movement science laboratories [15,32]. When assessed in the clinical setting, simple analog technologies [42] or subjective methods are commonly used. Reaction time, velocity, and other temporal aspects of movements are especially difficult to measure clinically due to the requirement of more advanced measurement devices. New sensor technology, including VR headsets, introduce novel possibilities for objective clinical assessments of sensorimotor functions and kinematics, similar to advanced laboratory equipment.

VR technology is used in several areas of physiotherapy and other medical fields with positive results on physiological, psychological, and rehabilitative outcomes [43,44]. The continuous development of VR technology is promising, with several diagnostic potentials, including development of new specific tests and exercises of cervical movement functions. In our previous study, VR showed good concurrent validity in measuring cervical range of motion (ROM) and movement velocity [45]. Other VR technologies have also been used for assessments of cervical kinematics, presenting acceptable validity and reliability [35,46,47,48].

In line with precision medicine, this gives new opportunities for more specific diagnostic tests, and thereby more precisely tailored and monitored treatment interventions in the clinic, and even at home. The VR headset in this study is equipped with a sensor that can measure movements in 3 degrees of freedom. The software is developed by Curest (Curest AB, Luleå, Sweden) and consists of built-in tests of cervical movement functions where the user interacts in the VR through head movements. The software has an online web-portal where test results are instantly uploaded. For clinical implementation of this new technology, it is important to evaluate the psychometric properties of the diagnostic tests, as well as the clinical feasibility and evidence of treatment effects of the interventions. This needs to be completed in separate steps. A first step is to evaluate the concurrent validity of the diagnostic test, i.e., compare new methods to a state-of-the-art measurement system. It seems reasonable to hypothesize that the VR technology is suited to assess specific sensorimotor functions of the cervical spine, including specific elements of proprioception such as position sense, and fast and accurate reaction movements of the head.

The aim of this study was to investigate the concurrent validity of a newly developed VR technology for assessment of neck sensorimotor function by comparing VR technology against a gold standard motion capture system. The sensorimotor tests evaluated were the established cervical joint position sense (JPS) test, and the novel cervical reaction acuity (CRA) test.

## 2. Materials and Methods

The study had a cross-sectional design. The measures from the VR headset (Curest AB, Luleå, Sweden) were compared to a gold standard 3-dimensional motion capture system (Qualisys, Gothenburg, Sweden) during simultaneous measures with both systems. Ethical approval was received through the Swedish Ethical Review Authority (ref No: 2022-00183-01). All participants gave written consent before participation in the study.

### 2.1. Participants

Participants were a convenience sample, recruited through advertisements at Luleå University of Technology, Sweden. Inclusion criteria were age 18–65, able to read and write in Swedish, being either non-symptomatic or having symptoms from the neck. The purpose of having a natural mix of neck status was to test the VR technology on a larger variety of neck functions. The objective was not to compare group differences between people with and without neck pain. The participants were seen as one group. Exclusion criteria were neck fracture, neck surgery, cervical radiculopathy, neurological disease, rheumatic disease, vestibular disease, uncorrected impaired vision, epilepsy, or previous experience of severe symptoms (nausea/dizziness) using VR glasses.

The sample size was based on power analysis with ICC values, where 0.6 was set as null hypothesis and 0.9 set to be statistically significantly higher than 0.6, *p* < 0.05 and 95% power. Based on this, 20 subjects were required [49].

### 2.2. Measurement Devices

The sensorimotor tests were performed using an immersive VR headset from Curest VR (Curest AB, Lulea, Sweden), software CurestVR_LTU-20221003, hardware Pico G2 4K (Pico Technology Co., Ltd., Cambridgeshire, UK). Immersive refers to the 3D virtual environment displayed in the glasses worn by the participants [50]. Curest VR uses a built-in Inertial Measurement Unit (IMU) comprising of accelerometer, magnetometer, and gyroscope to track movements in 3 degrees of freedom up to 100 Hz. In the VR environment, participants interact with objects on the screen, through their head movements. The movements are tracked by the IMU, and data are instantly transferred to an online portal where test results can be displayed. The weight of the VR headset is 276 g. It has adjustable straps for individual comfort, and regular glasses can be worn in the headset. The VR screen resolution is 3849 × 2160 pixels, with adjustable luminosity. The display has a bult-in, eye-protective blue-light-reducing system. A geomagnetic calibration was performed prior to each test session.

**Figure 1:**
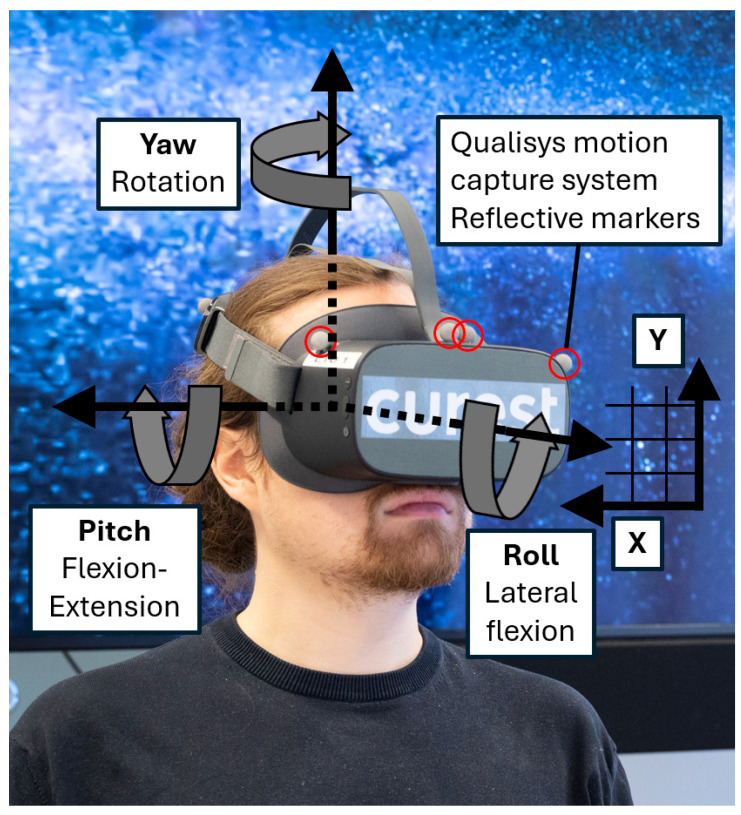
Experiment setup in the movement laboratory. Yaw, pitch, and roll are shown in Euler angles. X and Y are presented in Cartesian space. Co-author J.J. in photo, with consent.

The movements performed during the VR test were simultaneously measured with the Qualisys optoelectronic 3-dimensional motion capture system (8 camera, Oqus 400, Qualisys, Gothenburg, Sweden). Qualisys uses reflective markers attached to the moving object, in this case the VR headset. The reflective markers are detected by the cameras and movements are measured with very high accuracy, less than approximately 2 mm root mean square error [51,52], and can therefore be used as a gold standard system for 3D measurements [49,53]. The test setup is visualized in Figure 1.

### 2.3. Procedure

Tests and data collection were performed at the Human Health and Performance Lab—Movement Science at Luleå University of Technology, Sweden. Two tests were performed, the joint position sense (JPS), and the cervical reaction acuity (CRA) test.

The tests were instructed and supervised by an experienced registered physiotherapist (K.F.), and a laboratory technician (J.J.) was assisting with the data collection. The Numeric Rating Scale (NRS) 0–10 was used for self-rated neck pain intensity, where 0 represents “no pain” and 10 “worst imaginable pain”. NRS is a valid and reliable pain assessment instrument [54].

Participants were seated on a chair, 45 cm in height with an erect neutral position, back against the backrest and both feet on the ground. Instructions were to keep their back against the back rest, holding their trunk still and only moving the head during tests. A standardized warmup program for the neck region was performed without the VR headset. Before each test measurement, all participants performed one practice trial as familiarization. The VR screen was casted to a large screen enabling the test leader to follow the test procedure.

### 2.4. Outcome Measures

#### 2.4.1. Joint Position Sense (JPS) Test

**Figure 2:**
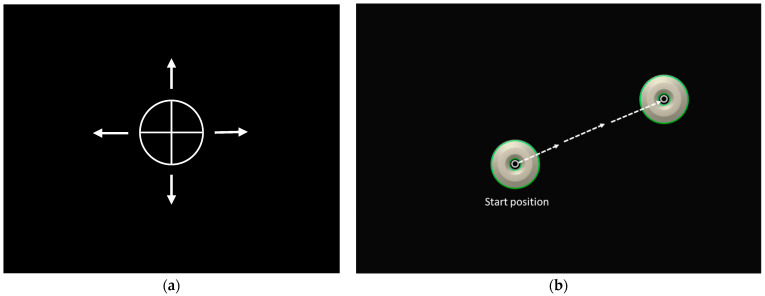
Example of the VR screen in the tests. (**a**) Joint position sense (JPS) test. An arrow indicated the movement direction the test was performed in for each direction: right rotation, left rotation, extension, and flexion, six trials in each direction. (**b**) Cervical reaction acuity (CRA) test. Participants started with the white circular marker aimed in the middle of the disc; the disc thereafter moved to a random place on the screen, whereby the participant moved their head as quickly as possible to the middle of the disc in the new location. The task continued to random places, 14 trials in total, always starting at the center of the screen.

In this test, the VR screen was dark, imitating being blindfolded. Participants were instructed to find their neutral head position, perform a head movement, and reposition their head back to the neutral position as accurately as possible (Figure 2a). The test was performed 6 times in each movement direction, right rotation, left rotation, extension, and flexion. Participants used a manual clicker device to indicate their neutral starting position and each time they were back in neutral. They were instructed to move as far as possible but in a comfortable range of motion at a self-selected speed. The initial neutral head position was the reference (target) point for the first movement direction that was right rotation. Thereafter, the last of the 6 repetitions in each movement direction served as reference position for the following movement direction. Absolute error (AE) in 2 dimensions, and variable error (VE) and constant error (CE) in 1 dimension, all in degrees (°) were used for data-analysis.

#### 2.4.2. Cervical Reaction Acuity (CRA) Test

Level 3 in Curest VR training protocol was used, representing a medium level of difficulty. The test started with the disc in the middle of the screen, participants keeping the head in neutral position with the circular marker aim in the middle of the disc (start position Figure 2b). During the test, the disc moved to random places on the screen. The task was to follow the disc as fast as possible by moving the head and stabilizing the head (aim) in the middle of the disk again for a second. The test comprised 14 repetitions in various unpredictable directions. Outcome measures were reaction time in milliseconds (ms) from the movement of the disc to the movement of the head, and maximum velocity in degrees per seconds (°/s) of the head movement.

### 2.5. Calculation of Outcome Variables

VR data were collected through the VR software CurestVR_LTU-20221003, (Curest AB, Luleå, Sweden). Data from the Qualisys motion capture system were collected through the software Qualisys Track Manager (QTM), version 2021.2, (Qualisys, Gothenburg, Sweden). For calculation of outcome variables, the Matlab program R2023, was used.

#### 2.5.1. JPS Test

At the start of the JPS test, the VR sensor was calibrated in the neutral head position and simultaneous event markers were made manually in VR and QTM by the test leader to synchronize the systems. Simultaneous event markers were also added manually every time the participant was back in neutral position, indicated by the participants clicker device.

For data from QTM measures, reflective markers on the VR headset were combined into a rigid body which was then exported to Matlab program R2023. Euler angles were taken from the exported file at the event markers. Curest data from the VR headset were exported from the platform with the individual repetitions for each task and person, assessed with the inbuilt IMU, in an excel file. For the first movement direction, origin was defined from the starting Euler angle position, while each subsequent direction had the origin defined as the Euler angle position of the last repetition of the previous movement direction.

Absolute error was calculated in degrees in 2 dimensions (D) as the 2-norm (Pythagorean theorem) of the flexion–extension (pitch) and right–left rotation (yaw) angles, Figure 1 (i.e., similar to the difference in target and reposition angle in a combined x and y direction on a 2D target in a Cartesian space). Constant error was calculated in 1D as the rotation direction over- or undershoot in degrees when rotating back from the end range of each movement direction. Variable error was calculated in degrees in 1D as the standard deviation of the constant error.

#### 2.5.2. CRA Test

To reduce noise in the velocity calculations, a Cartesian representation of the head direction was filtered with a 10 Hz Butterworth filter. Due to hardware limitations, the VR sensor gives measurements with uneven time steps. These were resampled from a cubic spline of the original VR measurements before the filtering to align with the QTM sampling frequency of 100 Hz.

Angular velocity was obtained by calculating the angle between each following measurement’s directional vectors and dividing by the measurement time window (10 ms). It was then filtered again with both a 10 Hz Butterworth filter and a moving average with a window of 100 ms to smooth out the roughness of the velocity peaks. The highest angular velocity in degrees/seconds (°/s) during the repetition was then set as maximum speed.

Reaction time in milliseconds (ms) was defined as the time from when the disc started to move, to the timepoint when the participant reached 5% of their maximum speed for the specific repetition. Since the start time is defined from the VR point of reference, the QTM data were shifted timewise so that the velocity peaks for the first repetition aligned. Most velocity noise was mitigated with the filtering, but for four participants the end of the reaction time window was manually adjusted from visual assessment to better represent the start of the movement.

### 2.6. Statistics

The Statistical Package of Social Sciences (SPSS), version 28.0.0.0 (190) (IBM, Armonk, NY, USA) was used for statistical analyses. Assumptions for data variables for each statistical test were checked through histogram, Q-Q plots, skewness and kurtosis values, and the Shapiro–Wilk test. Significance level was set at ≤0.05. Concurrent validity was analyzed through the Bland–Altman method which gives information about agreement between measures, i.e., VR and Qualisys. The method uses the differences between the two systems (Qualisys minus VR) plotted against the mean of both measures. The mean difference (mean bias) is presented together with 95% Limits of agreement (LOA) being [mean bias ± (1.96 × standard deviation)] [55,56]. The mean difference was also calculated to a percentage value with the formulae [(mean difference Qualisys − VR)/mean of both systems] × 100. To analyze the correlation between the two measurement systems, the Intraclass Correlation Coefficient (ICC; 2.*k*) two-way random effects, absolute agreement, average measures was used [57]. Reference values for the ICC are <0.5 poor, 0.5–0.75 moderate, 0.75–0.9 good, and >0.90 excellent correlation [57]. The paired samples *t*-test was used to analyze the difference between the VR and Qualisys data for any systematic bias.

## 3. Results

The 20 participants (10 women and 10 men) performed all the tests successfully. Participants’ background information is presented in Table 1. Seven participants had current neck pain with a mean NRS rating of 2.6 (1.1). Thirteen participants were asymptomatic, i.e., no neck pain.

### 3.1. Joint Position Sense Test

**Figure 3:**
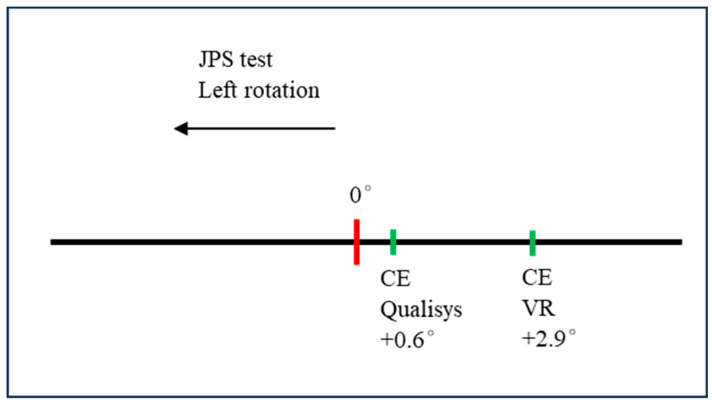
JPS test, CE (one dimension) after left cervical rotation. These positive values represent “overshooting”, i.e., crossing zero.

The JPS mean values from Qualisys and VR together with the paired samples *t*-test analyses are presented in Table 2. In general, the mean differences were small (mean ≤ 0.7°) for all directions except rotation left. For rotation left, AE, CE, and VE were significantly different between Qualisys and VR, with the largest mean difference for CE rotation left (−2.4°). The CE variables show a slight overshooting in all directions except extension. Positive CE values represent “overshooting”; an example is provided in Figure 3.

**Figure 4:**
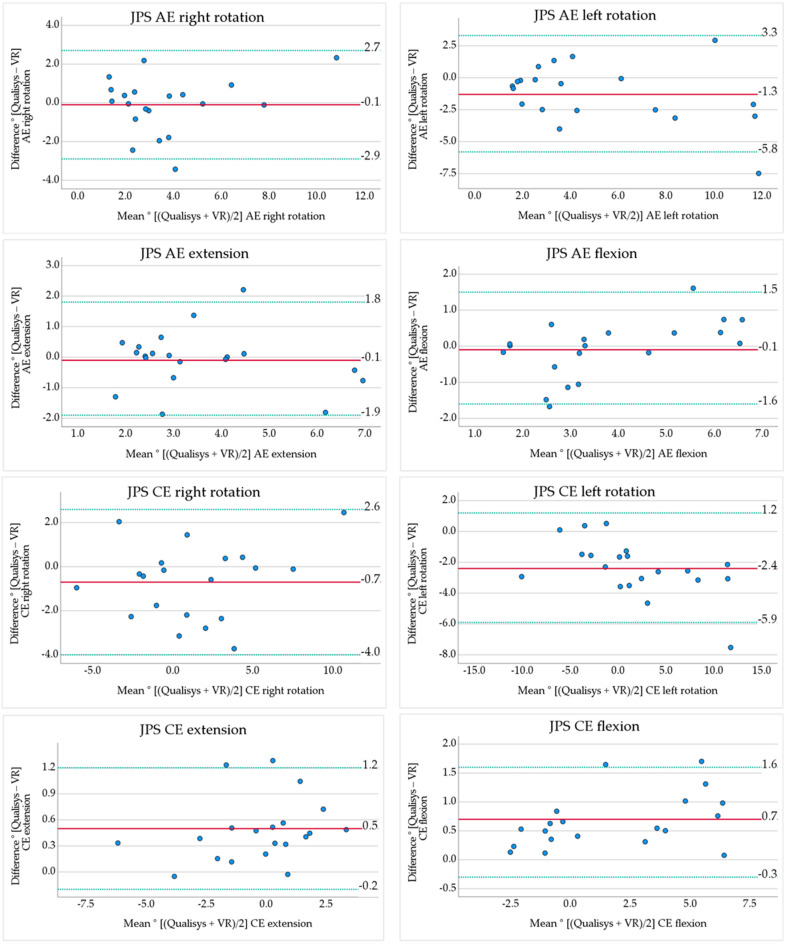

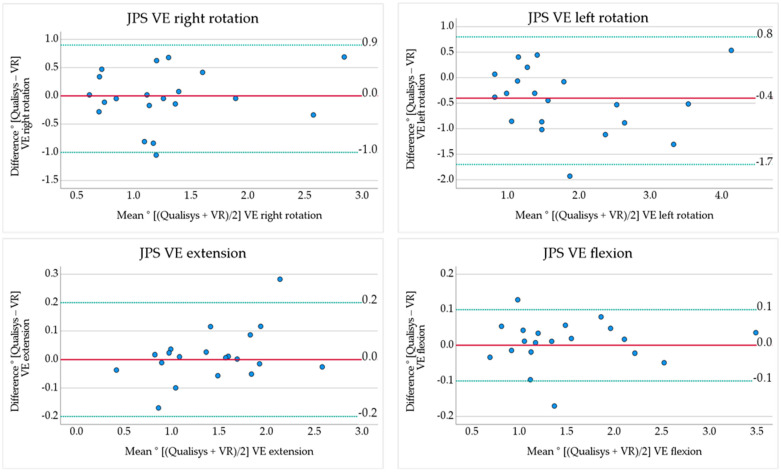
Bland–Altman plots of JPS test variables. The y-axis contains the difference Qualisys minus VR, the mean difference (red line), and 95% LOA (green dotted lines). X-axis contains the mean value of Qualisys and VR.

Absolute error (AE) had a mean bias of −0.1° (representing −2.6–−2.8% mean bias as percent of the mean values) in all directions except left rotation with a mean bias of −1.3° (−25.2%) (Table 3 and Figure 4). All the AE variables had negative mean bias values, meaning VR results were slightly larger compared to Qualisys. This was significant only for rotation left (Table 2).

Constant error (CE) revealed mean bias values <0.7° except left rotation with −2.4° (LOA −5.9° to 1.2°) (Table 3 and Figure 4). The mean bias in percent ranged from 38.9 to 166.7%. Significant differences were shown for rotation left, extension, and flexion, with larger values for VR in left rotation and extension (Table 2).

Variable error (VE) revealed almost no mean bias between the systems (Table 3 and Figure 4). Although, VE for left rotation had a relatively large LOA −1.7° to 0.8° (Table 3 and Figure 4) and was significantly different between measurement systems (Table 2). The mean bias in percent was 0% except VE left with −21.6%.

The ICC results are presented in Table 3. ICC for JPS test variables were all significant, (*p* < 0.001) and ranged between 0.835 and 0.998 indicating a good to excellent correlation between VR and Qualisys.

### 3.2. Cervical Reaction Acuity Test

The mean differences between VR and Qualisys for reaction time and maximum velocity are presented in Table 4.

**Figure 5:**
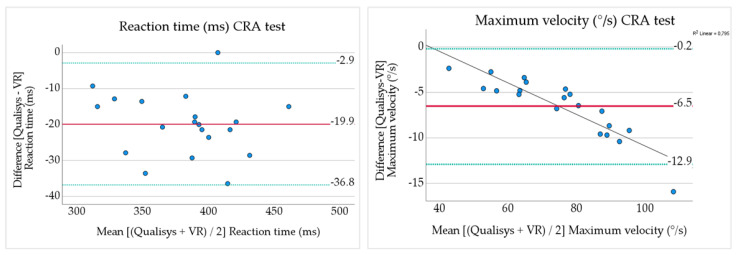
Bland–Altman plots of reaction time and maximum velocity from Cervical reaction acuity test. The y-axis contains the difference Qualisys minus VR, the mean difference (solid red line), and 95% LOA (green dotted lines). The x-axis contains the mean value of Qualisys and VR. In the plot for maximum velocity, a regression line (black line) is presented to illustrate the downward trend with increased difference as velocity increased.

Reaction time had a mean bias −19.9 ms (LOA −36.8–−2.9 ms) (Table 5 and Figure 5) representing −5.2% of the mean values (Table 5). This bias was systematic with slightly higher values for the VR system (Table 4).

Maximum velocity also showed a small bias, −6.5°/s (LOA −12.9–−0.2), with VR giving slightly higher values compared to Qualisys (Table 5 and Figure 5). The mean bias in percent was −8.7%. Due to the linear downward trend seen in Figure 5, we also performed a Pearson correlation analysis of the maximum velocity difference variable against maximum velocity values (mean of Qualisys and VR). This result showed Pearson r −0.89, *p* < 0.001.

ICC values were 0.931 for reaction time and 0.954 for maximum velocity indicating excellent correlation.

## 4. Discussion

The aim of the study was to investigate the concurrent validity of the VR-based JPS test and the novel CRA test in a group of people with and without neck pain. All JPS variables showed small differences (<0.7°), except left rotation. Left rotation stood out with mean differences in AE −1.3°, CE −2.4°, and VE −0.4°. A good to excellent correlation ICC 0.835–998 was observed for all JPS variables. From the CRA test, reaction time showed a small mean bias of −19.9 ms. Also, maximum velocity showed a small mean bias of −6.5°/s. Reaction time and maximum velocity had ICC values 0.931 and 0.954, respectively, indicating an excellent correlation between VR and Qualisys. The results from this study show an acceptable concurrent validity of the VR technology for assessment of the JPS and the CRA test.

Our results are in line with previous research on concurrent validity of the JPS test. The validity of the JPS test has been evaluated using a customized VR system comprising laser-emitting lighthouses and trackers by comparison to the VICON motion capture system. The study reported the root mean square error of the JPS test between the systems to be ≤2.2° [58]. The JPS test with laser pointer has been correlated with an ultrasound device, r > 0.9 [30], and the Fastrak system, r > 0.8 [31], which is similar to our results regarding correlations (ICC). These results can, however, not be fully compared to our results due to different technologies and analysis methods.

Although clinical methods for assessment for cervical JPS have been available for three decades [59], few studies have evaluated the concurrent validity of these clinical tests. In particular, there is a lack of studies using both ICC and the Bland–Altman method, which have been argued to be the preferred methods for clinical tests and diagnostics [55]. To the best of our knowledge, no other studies have investigated the JPS test in a VR headset and used the Bland–Altman method and ICC against gold standard for analysis of concurrent validity. Christensen et al. [60] used an IMU device (MOTI) to measure the JPS test and studied concurrent validity by comparison to the Optotrack Certus 3D camera system. They found a mean bias in AE right rotation −0.4° (LOA −1.9 to 1.09°) and AE left rotation −0.56° (LOA −2.02 to 0.9°). This is in line with our results, although our results show a slightly higher mean bias in left rotation with higher LOA.

The AE is probably the most used outcome variable in the JPS test in research and clinical work. This is also relatively easy to calculate with the analog laser pointer technique and gives a general measure of the error. In our study, with a mixed group of individuals with and without neck pain, the participants had a mean AE of 3.6–5.8° measured with the VR system. This is in line with normative values being <4.5° for healthy persons [27,30]. For future research, we also presented data for CE and VE, which represent other aspects of position sense. The CE represents a systematic bias of over- or undershooting, while VE represents the precision, or consistency, of the task at hand [19].

In the CRA test, the reaction time and maximum velocity were measured. These variables have been measured in previous studies with various technologies, including VR but in different test set-ups [39,40,48]. However, there seems to be a lack of studies evaluating the concurrent validity of VR headsets for reaction time and velocity in fast movement tests. An IMU device has been evaluated for concurrent validity of movement velocity during a cervical range of motion test by comparison to an optoelectronic system [61]. The study found mean differences of around 4–11°/s and LOA up to ±20°/s. The mean difference is in line with our results (−6.5°/s) but our LOA were smaller (±6.4°/s). The Bland–Altman plot for maximum velocity (Figure 5) showed a negative linear correlation meaning larger differences between the two systems as the velocity increased. This was not seen in the maximum velocity variable from the fast cervical rotation test in our previous study for the same velocity range. Although, it was seen in very high velocities, above 550°/s [45]. These errors should be noted and further investigated in future studies.

The reaction time of our CRA test showed a mean value of 392.3 (40.9) ms measured with VR. This is slightly higher compared to previous reported values from asymptomatic people, mean 260–290 ms [39], and median ≈ 320 ms [40], and lower compared to patients with whiplash-associated disorders, mean 410–430 ms. Considering this present study’s composition of participants both with and without neck pain, our reaction times seem reasonable. The comparative studies [39,40] used different test set-ups and Ohberg et al. [39] calculated the reaction time slightly different from us which may be other explanations for the discrepancy in normative values from our study. In Ohberg et al. [39], the reaction time was measured with a motion tracking system during a test where the task was to move the head as fast as possible in right or left rotation, flexion, or extension indicated by an arrow on a board in front of them, the arrow giving random movement direction indications. Gadotti et al. [40] had a task where participants reacted to targets appearing on a screen, simulating driving a car and reacting to events.

The mean maximum velocity in the CRA test measured with VR was 78.2 (18.2)°/s. This can be compared to a similar test performed in another VR headset, where the task was to steer a plane with head movements to a target appearing in random locations on the VR screen. Maximum velocities were 149–262°/s for asymptomatic people [35]. An earlier VR study from Sarig Bahat et al. showed the maximum velocity in asymptomatic people to be 105–166°/s, where the task was similar, to move the head quickly to targets [48]. Our study demonstrates lower values compared to these studies. However, since the maximum velocity is related to, e.g., the range of the head movement, and since the task specifics were different between the studies, further research is necessary to establish normative data. Our study also included a mix of people with and without neck pain, which is a possible explanation for lower values in maximum velocity. For clinical assessment of reaction time and maximum velocity of cervical movements, it is desirable to have standardized tests. The psychometric properties of the proposed CRA test in this study need to be further evaluated, e.g., for construct (known-group) validity and reliability.

Some findings should be highlighted for further investigation and use of the methodology. The results from the JPS test give indications of a slight drift of the VR measurements in the transverse plane, i.e., rotation right and left. Left rotation stood out as having the largest mean bias in AE, CE, and VE, where CE had a mean difference of −2.4° (LOA −5.9 to 1.2) compared to the Qualisys motion capture system, meaning a drift of 2.4° to the right in VR. A similar drift was seen in the same motion plane for rotation right during the range of motion test in the same VR technology used in our previous study [45]. The actual reason is not known but could be an artificial drift in the VR as discussed in Forsberg et al. [45] and reported in an earlier VR study [62]. The mean bias percentage of the CE is distinctly larger than for the other variables in our study. This is related to the fact that the mean bias is large in relation to the mean values, since there are both positive and negative values with mean values close to zero. The use of this variable may be questionable and should be evaluated in future research on construct validity and reliability of the test.

There are some limitations in this study. The JPS test was developed with inspiration from previous studies [30,31,63]. In these studies, they performed a passive reposition of the participant’s head between each repetition to ensure the same reference position for all repetitions without giving participant feedback. The passive repositioning was not possible in our study; instead, the first neutral position served as reference position for all right rotation repetitions. Thereafter, the patient’s last neutral position from each previous movement direction served as reference position for the next movement direction. This may be a weakness as the exact target position was not reset before each repetition.

We did not use straps for thorax to reduce movements of the chest during movements. However, we used a chair with a back rest and visual inspection to standardize the test and minimize movements from the thorax or shoulders.

This current study included 20 participants which can be considered a small sample. This can have implication for the limits of agreement as single extreme values may affect results. Construct validity, including differences between people with neck pain and asymptomatic people, reliability, and responsiveness of VR-based sensorimotor tests should be researched in future studies, including larger samples. The tests were performed in a laboratory setting with guidance from a physiotherapist. This may differ from the home-based use where patients may perform tests on their own. This should also be investigated in future studies.

### Clinical Implications

This study adds important results regarding concurrent validity of the JPS and CRA tests performed in the Curest VR environment, where both tests show acceptable biases compared to gold standard. This has clinical implications since position sense, reaction time, and movement velocity can be reduced in people with neck pain [15,27,28,29,30,31,35,38,39,40,41]. In particular, reaction time and velocity have traditionally been difficult, or impossible, to measure with the standard clinical analogue methods. Feasible clinical methods for objective assessments of movement functions are important to identify specific movement disturbances for precise tailored interventions and evaluation of treatment effects, both in clinics and research. It should be noted that the result from this study is applicable for the specific hard- and software used in this study and may not be generalized to all VR systems as they might use other tracking technologies and algorithms.

## 5. Conclusions

The results from this study show acceptable concurrent validity of the VR technology in measuring JPS errors and CRA reaction time and movement velocity. A slight bias was observed in left rotation in the JPS test, as well as higher values for reaction time and velocity in the CRA test with the VR technology. This should be considered and further explored in future research. Moreover, future research should investigate the construct (known-group) validity, repeatability (reliability), and responsiveness of the proposed tests.

## Figures and Tables

**Table 1:** Participant characteristics.

	Mean ± SD
Age (years)	45 ± 12
Weight (kg)	82 ± 17
Height (cm)	174 ± 10

SD: standard deviation; kg; kilogram; cm: centimeter.

**Table 2:** JPS test. Mean values (SD) from Qualisys and VR. Paired samples *t*-test. VR compared to Qualisys.

JPS Variables	Qualisys Mean (SD)°	VR Mean (SD)°	Mean Difference (SD)°	Std. Error Mean°	95% Confidence Interval of the Difference		t	Two-Sided *p*
					Lower	Upper		
**AE right**	3.6 (2.6)	3.7 (2.3)	−0.1 (1.4)	0.3	−0.8	0.6	−0.3	0.741
**AE left**	4.5 (3.4)	5.8 (4.3)	−1.3 (2.3)	0.5	−2.3	−0.2	−2.4	0.025
**AE extension**	3.5 (1.5)	3.6 (1.7)	−0.1 (0.9)	0.2	−0.5	0.4	−0.4	0.714
**AE flexion**	3.8 (1.9)	3.8 (1.5)	−0.1 (0.8)	0.2	−0.4	0.3	−0.4	0.719
**CE right**	1.0 (4.2)	1.7 (3.9)	−0.7 (1.7)	0.4	−1.5	0.1	−1.8	0.082
**CE left**	0.6 (5.5)	2.9 (6.5)	−2.4 (1.8)	0.4	−3.2	−1.5	−5.9	<0.001
**CE extension**	−0.1 (2.3)	−0.5 (2.2)	0.5 (0.4)	0.1	0.3	0.6	5.8	<0.001
**CE flexion**	2.1 (3.4)	1.5 (3.2)	0.7 (0.5)	0.1	0.4	0.9	6.3	<0.001
**VE right**	1.3 (0.7)	1.3 (0.6)	0.0 (0.5)	0.1	−0.3	0.2	−0.3	0.805
**VE left**	1.6 (1.0)	2.1 (1.0)	−0.4 (0.6)	0.1	−0.7	−0.1	−3.1	0.006
**VE extension**	1.4 (0.6)	1.4 (0.5)	0.0 (0.1)	0.0	0.0	0.1	0.7	0.522
**VE flexion**	1.5 (0.7)	1.5 (0.7)	0.0 (0.1)	0.0	0.0	0.0	0.5	0.643

AE: absolute error; CE: constant error; VE: variable error; SD: standard deviation; JPS: joint position sense; °: degrees.

**Table 3:** JPS test VR compared to Qualisys. Bland–Altman mean bias and 95% LOA, Intraclass Correlation Coefficient and 95% CI.

JPS Variables	Mean Bias° (Qualisys − VR)	95% LOA°		Mean Bias %	ICC_2.*k*_	ICC 95% CI	
		Lower	Upper			Lower	Upper
AE right	−0.1	−2.9	2.7	−2.7	0.911 ***	0.775	0.965
AE left	−1.3	−5.8	3.3	−25.2	0.879 ***	0.655	0.954
AE extension	−0.1	−1.9	1.8	−2.8	0.911 ***	0.774	0.965
AE flexion	−0.1	−1.6	1.5	−2.6	0.945 ***	0.862	0.978
CE right	−0.7	−4.0	2.6	−51.9	0.948 ***	0.864	0.980
CE left	−2.4	−5.9	1.2	−137.1	0.940 ***	0.266	0.985
CE extension	0.5	−0.2	1.2	−166.7	0.983 ***	0.688	0.996
CE flexion	0.7	−0.3	1.6	38.9	0.985 ***	0.645	0.996
VE right	0.0	−1.0	0.9	0.0	0.835 ***	0.580	0.935
VE left	−0.4	−1.7	0.8	−21.6	0.846 ***	0.480	0.945
VE extension	0.0	−0.2	0.2	0.0	0.993 ***	0.982	0.997
VE flexion	0.0	−0.1	0.1	0.0	0.998 ***	0.995	0.999

*** *p* < 0.001; ICC: Intraclass Correlation Coefficient; 2.*k*: two-way random effects model, absolute agreement, average measures; CI: Confidence Interval; AE: absolute error; CE: constant error; VE: variable error; Mean bias°: the mean difference in degrees between Qualisys and VR measurements (Qualisys minus VR); LOA = Limits of agreement. Negative mean bias values represent higher values for the VR device whereas positive values represent lower values for VR; Mean bias %: the mean difference between Qualisys and VR measured as a percentage of both systems [(mean difference Qualisys − VR)/mean of both systems] × 100.

**Table 4:** Cervical reaction acuity test. Mean values (SD) from Qualisys and VR. Paired samples *t*-test. VR compared to Qualisys.

Cervical Reaction Acuity Test Variables	Qualisys Mean (SD)	VR Mean (SD)	Mean Difference (SD)	Std. Error Mean	95% Confidence Interval of the Difference		t	Two-Sided *p*
					Lower	Upper		
Reaction time (ms)	372.5 (39.4)	392.3 (40.9)	−19.9 (8.6)	1.9	−23.9	−15.8	−10	<0.001
Max velocity (°/s)	71.7 (15.3)	78.2 (18.2)	−6.5 (3.3)	0.7	−8.1	−5.0	−9	<0.001

SD: standard deviation; ms: milliseconds; °/s: degrees per seconds.

**Table 5:** Cervical reaction acuity test. VR compared to Qualisys. Bland–Altman bias and 95% LOA. Intraclass Correlation Coefficient and 95% CI.

Cervical Reaction Acuity Test Variables	Mean Bias (Mean Difference Qualisys − VR)	95% LOA		Mean Bias %	ICC_2.*k*_	ICC 95% CI	
		Lower	Upper			Lower	Upper
Reaction time (ms)	−19.9	−36.8	−2.9	−5.2	0.931 ***	−0.069	0.986
Max velocity (°/s)	−6.5	−12.9	−0.2	−8.7	0.954 ***	0.014	0.991

ms: milliseconds; °/s: degrees per seconds; *** *p* < 0.001; ICC: Intraclass Correlation Coefficient; 2.*k*: two-way random effects model, absolute agreement, average measures; CI: Confidence Interval; Mean bias: the mean difference between Qualisys and VR measurements (Qualisys minus VR); LOA = Limits of agreement. Negative mean bias values represent higher values for the VR device whereas positive values represent lower values for VR; Mean bias %: the mean difference between Qualisys and VR measured as percentage of both systems [(mean difference Qualisys − VR)/mean of both systems] × 100.

## Data Availability

The data presented in this study are available on request from the corresponding author.
